# Supplementary material for: Are carbon emissions trading and green financial instruments synergistic? -Comprehensive quantitative research based on content analysis
Source: PLoS One. 2024 Mar 7;19(3):e0298601. doi: 10.1371/journal.pone.0298601 (PMC10919601; doi:10.1371/journal.pone.0298601)
Supplement: S1 Data — (ZIP) [file pone.0298601.s001.zip › Data/地级市Z_F工作报告.zip!/╜¡╦╒/─╧═¿╩╨/└┤╫╘╣1⁄2╓┌║┼í╛┬φ┐╦╩2╛▌═°í┐_2012.docx]

各位代表：

　　现在，我代表市人民政府向大会作工作报告，请予审议，并请市政协委员和其他列席人员提出意见。

　　一、2011年工作回顾

　　2011年是“十二五”开局之年。我们在省委、省政府和市委的正确领导下，深入贯彻落实科学发展观，抢抓国家战略机遇，统筹推进各项工作，较好完成了市十三届人大四次会议确定的目标任务，实现了“十二五”良好开局。预计，地区生产总值达到4100亿元，按可比价计算比上年增长12%；财政总收入951.7亿元，其中，地方一般预算收入373.69亿元，增长28.5%；固定资产投资2380亿元（新口径），增长21.7%；社会消费品零售总额1480亿元，增长17%；外贸进出口总额250亿美元，增长18.5%；注册外资实际到账25.45亿美元；城镇居民人均可支配收入25100元，农村居民人均纯收入11500元，分别增长15%和16%；居民消费价格指数涨幅低于全省平均水平；城镇登记失业率2.8%；全社会研发投入占地区生产总值比重2.05%；基本完成节能减排目标任务。

　　我们全面贯彻市委“落实‘八项工程’、实现‘八个领先’”要求，加强重大问题调研，目标化、项目化推进重点工作，整体展开“十二五”工作布局。全力应对复杂多变形势，出台千方百计保持经济平稳较快发展等政策措施，牢牢把握经济工作主动权。我市成功蝉联全国文明城市称号，通过国家卫生城市、国家园林城市复查验收。

　　我们主要做了以下工作：

　　（一）以转型升级促进经济又好又快发展。落实转型升级和科技创新工程，推进产业升级“三大计划”，着力构建以先进制造业为主体、现代服务业为支撑、现代农业为基础的产业新格局。

　　全力提升主导产业。推动主导产业“双倍增、双突破、双提升”，制定加快市区工业经济和纺织服装等产业转型升级的意见，实施质量强市战略，六大主导产业产值全部突破千亿元。我市跻身世界三大家纺产品集散中心。国家海洋工程装备与船舶产业集聚标准化示范区获批启动建设。建筑业施工总产值达到3200亿元，新增鲁班奖4个，10家企业进入全国百强建筑企业行列。

　　突出发展新兴产业。按照基地化发展方向，实施新兴产业发展“310”工程，出台创建特色产业基地等政策，推进10个特色产业基地建设，新兴产业产值突破2000亿元。制定现代服务业提速发展三年行动计划，出台加快服务业发展和现代服务业集聚区建设等政策意见，服务业增加值占地区生产总值比重达到38.5%。

　　大力发展现代农业。落实农业现代化工程，放大高效农业规模优势，高效农业面积占全部耕地比重突破50%，粮食生产实现“八连增”。制定实现农业产业化发展全省领先的意见，新增省级现代农（渔）业园区4家、国家级和省级农（渔）业产业化龙头企业15家，省级现代农业园区实现县（市）区全覆盖。

　　着力推进科技创新。实施加快建设创新型城市意见，实现全国科技进步先进市（县、区）“满堂红”。实施新一轮“高新技术产业双倍增”工程，高新技术企业达到280家，产值突破3000亿元。推动自主型、开放式创新，中远船务“深海高稳定性圆筒型钻控储油平台的关键设计与制造技术”获国家科技进步一等奖，我市通过国家知识产权示范市验收；实施产学研合作项目349个，产业技术创新战略联盟总数达到16家；新增科技投融资机构9家，初步构建“四位一体”科技金融服务体系。推动创新载体建设，通州开发区获批为省级南通高新区，中科院南通光电工程中心等重大研发机构启动建设，新增省级企业院士工作站9家、企业技术中心12家、公共技术服务平台3家。深入实施“江海英才”计划，引进领军型人才和紧缺专业优秀人才2000多名，新增国家“千人计划”人才2名、省“双创计划”人才（团队）18名。

　　（二）以项目建设推动沿海开发提速。

　　推动港口和通道加速建设。实施22个重大港口和通道项目，完成投入超过100亿元。洋口港区中石油江苏LNG项目投产，10万吨级航道建成，10万吨级石化码头开工；吕四港区10万吨级挖入式港池基本完成前期论证，东灶港作业区建设进展顺利；通州湾综合开发前期工作快速推进。沿江14个码头扩能升级。崇启大桥通车，平海公路东段建成，通洋高速、临海高等级公路加快推进。海洋铁路基本建成，沪通铁路开工准备工作深入推进，宁启铁路复线电气化改造进展顺利。兴东机场飞行区改扩建工程竣工。连申线航道整治快速推进。

　　推动产业项目加速集聚。全力推进170个市级重点项目和100个沿海前沿区域重大项目，组织10多次项目集中开工。一批单体总投资超50亿元的重大项目落户，百亿元级项目开工。

　　推动载体功能加速提升。实施加快开发区转型升级意见，省级以上开发区配套功能和集聚能力不断增强。推进跨江合作开发，苏通科技产业园8个超亿美元产业项目开工，研发中心等配套项目竣工；锡通科技产业园正式挂牌，专项配套政策出台。推动海关特殊监管区转型升级，南通综合保税区申报工作全面启动。8个沿海前沿镇实施“区镇合一”管理体制。全市围垦滩涂10.3万亩。一批功能性项目加快建设。

　　（三）以改革开放增强发展活力。

　　加快开放型经济转型升级。大力实施“六千六百六十工程”，对外开放水平进一步提高。加大招商选资力度，突出沿海开发、新兴产业、现代服务业等主题，举办港洽会，组织赴欧美、日本、港台、上海、深圳等地开展系列招商活动，引进超3000万美元项目43个。扩大优势产品出口，我市成为全国首批国家船舶出口基地，24个出口品牌成为省重点培育发展的国际知名品牌。保持外经领先优势，完成对外承包劳务营业额14亿美元，新批境外投资项目50个，中方协议投资额4.5亿美元。服务外包营业额增长75%，崇川区科技园成为中国电子商务创新发展示范基地。外事、侨务、港澳事务、对台事务、口岸工作在扩大开放中发挥了积极作用。

　　加快民营经济扩量提质。积极争创江苏民营经济第一大市，民营经济增加值、税收和民间投资占全市比重分别达到61%、65%、74%。深入推进全民创业，个体工商户数、私营企业数分别增长15.9%、21.2%，引进市外民资注册资本突破600亿元。大力推动集约发展，新增百亿级特色板块2个。切实抓好“三名”工程，20家企业入选全国500强民营企业；新增省级以上名牌产品68个，驰名、著名商标33件。

　　加快重点领域改革。推动金融体制改革，新设6家村镇银行和1家股份制银行，首家外资银行日本名古屋银行南通分行开业，银行业金融机构总数达34家；企业直接债务融资73.4亿元；新增担保机构、小贷公司16家；新增上市公司5家，2家上市公司再融资，共募资92.1亿元。国有资产实现保值增值。推进事业单位绩效工资制度改革。启动经济发达镇行政管理体制改革试点工作。

　　（四）以统筹城乡发展加快一体化进程。

　　大力度推进中心城市建设。按照建设长三角北翼经济中心的定位，制定加快中心城市建设意见，促进中心城市功能完善、品位提升。完善规划体系。完成中心城市空间发展战略研究、新城区拓展概念规划和各类专项规划等24项规划，金融街区、五山地区、通吕运河等重点区域规划研究全面展开。加强重点区域建设和改造。实施117个重点城建项目，完成投资180亿元。新城区一批商贸、金融、文化、教育、医疗等功能性项目加速推进。观音山新城启动建设。开发区能达商务区一批功能配套项目竣工。老城区历史街区和伶工学社等历史文化保护工程启动。北翼新城核心区建设提速。通州城区开发步伐加快。推进市区“102030”交通畅通工程。长江中路、江海大道、通宁大道3条高架通车，“一环一轴八射”快速路网初具形象；静海大桥、世纪大道东延等骨干路桥工程竣工；工农路公交专用道建成使用。强化城市科学化管理。完成市区城中村改造、老小区整治、农贸市场升级等项目40多个；加强环境管理，市容形象出新；增强为民服务功能，新增绿地200公顷，完成西气东输工程天然气置换近15万户。

　　大力度推进重点城镇建设。完善城镇规划体系，县（市）城、乡镇总体规划修编全面展开。支持各县（市）城做大做强，5个县（市）城完成城建投入超过100亿元。加强滨江临海新城、中心镇建设。全市城市化率达57.5%，提高1.5个百分点。

　　大力度推进新农村建设。加大强农惠农力度，推动农村改革发展，新增农村三大合作组织入社成员93.6万户。加快推进水利现代化建设，完成水利建设投入19.8亿元。优化农村发展环境，农村和市郊“四位一体”环境长效管理不断深化，村庄环境整治启动，新增高标准农田21.1万亩、植树造林面积17.5万亩。改善农村公共服务，新建农村公路1244公里，新建改造农村公路桥梁和农用桥梁861座；新增区域供水受益人口70万人。制定推进村级“四有一责”建设行动计划和扶持经济薄弱村发展的意见。

　　大力度推进生态文明建设。落实生态文明建设工程，海安成为全省江北首个国家生态县，其他四县（市）及通州区均达到国家生态县考核标准。加强环境保护，大力实施蓝天、清水工程，空气质量得到改善。推进资源节约，全面实施新一轮土地利用总体规划，连续20年保持耕地占补平衡；新建12个秸秆能源化利用示范点；通过国家级节水型社会试点市建设中期评估。

　　（五）以加强社会建设提升公共服务水平。扎实推进民生幸福工程，制定加强社会建设构建民生幸福城市的意见，全市财政民生投入占一般预算支出比重达到55%；10件40项政府为民办实事项目全面完成。

　　增加城乡居民收入。实施城乡居民收入倍增计划，推动创业富民、就业富民，“万人创业计划”全面启动，全市新增城镇就业8.65万人，81%的村建成“充分就业村”；拓宽物业、投资等富民渠道，城乡居民财产性、经营性收入明显增加。

　　提高社会保障水平。完善城乡社会保障体系，提高新农合筹资、城乡低保等保障标准和城镇职工、居民医保报销比例，出台城镇居民社会养老保险意见，启动城镇职工基本医疗保险和生育保险市级统筹，建立市区被征地农民基本生活保障与城镇企业职工基本养老保险衔接制度。强化保障性安居工程建设，市区拆迁安置房竣工交付超200万平方米，安置拆迁户超1万户，保障性（限价）商品房开工并具备预售条件超6000套，实现新建住房价格控制目标。扎实推进“菜篮子”工程建设，南通农副产品物流中心一期工程蔬菜、果品交易区建成，建设5万多亩蔬菜保供基地，设立50家平价商店。

　　加快社会事业发展。落实教育中长期改革和发展规划纲要，实施中考改革和高职教育综合改革试点项目，推进中小学校舍安全工程、学前教育普及提升和基础教育优质均衡发展，职业教育、高等教育服务地方发展能力增强。强化城乡公共卫生和医疗服务，医改三年任务基本完成，基本药物制度实现镇、村全覆盖。落实文化建设工程，公共文化服务体系加快构建。加快推进市图书馆新馆、报业新闻传媒中心等文化设施项目，筹建中华慈善博物馆，环濠河博物馆群被列为国家首批公共文化服务体系示范创建项目；有线数字电视实现城乡全覆盖。全民健身、社会养老、计划生育等服务水平提高。职工权益维护、妇女、儿童、青少年、残疾人、关心下一代、老区扶贫开发、见义勇为、法律援助、红十字和慈善等工作进一步强化。民族、宗教、新闻出版、哲学社会科学等工作不断加强。

　　（六）以社会管理创新保持社会和谐稳定。落实社会管理创新工程，扎实推进社会管理创新综合试点工作，我市以“三先四化”为统领构建社会管理创新“八大体系”的先行先试经验获得中央肯定。完善社会管理机制。注重从源头预防和化解社会矛盾，创新发展“一综多专”大调解体系，完善医患、劳资等专业调处机制，全面推行社会稳定风险评估、群众利益维护等制度。夯实基层基础。推进“强基工程”，基层社区、政法综治、群团社团等组织作用进一步发挥，社会管理基础工作得到加强。深化长安南通、法治南通创建。全市万人发案率保持全省最低水平，公众安全感继续位居全省前列，安全生产事故起数和死亡人数连续10年双下降。应急管理体系不断健全，食品安全检测体系进一步完善，食品药品安全监管扎实有效。我市被评为全国“五五”普法先进市；国防教育、民兵预备役、国家安全、防邪反邪等工作取得新成效，实现全国双拥模范城“五连冠”。

　　（七）以效能提升推动政府自身建设。把提高效能作为做好各项工作的重要保障，构建服务型政府取得新成效。推动效能提速。全面推行行政审批“三集中、三到位”工作，取消涉企行政事业性收费31项。增强行政执行力，完善政府工作推进、协调、督查三大体系，120项重点工作全面完成。强化依法行政。切实加强法治政府建设，健全重大事项决策机制。全面清理规范性文件，营造良好政策环境。扩大政府信息公开范围，提高行政权力网上公开透明运行水平。自觉接受市人大及其常委会的法律监督、工作监督和市政协的民主监督，认真执行人大及其常委会决议，支持政协参政议政。对市人大“坚持规划领先，强化组织协同，推进南通沿海一体化开发”的代表议案和市政协“加快培育和发展我市新兴产业”、“加快我市养老服务体系建设”的建议案，专题交办、专人跟办、专项督办，促进了重点工作落实。全面完成市人大代表建议、政协提案办理任务。加强廉政建设。对照《廉政准则》，推进制度建设，规范权力运行，初步建成政府系统惩防体系基本框架。以政务诚信引领社会诚信建设，落实《南通市公务员思想道德和诚信行为规范》，公务员队伍建设得到加强。切实加强审计监督。领导干部廉洁自律意识进一步增强。

　　各位代表，过去一年所取得的成绩，是省委、省政府和市委正确领导的结果，是全市人民和衷共济、奋力拼搏的结果。在此，我谨代表市人民政府，向广大干部群众，向人大代表、政协委员和离退休老同志，向市各民主党派、工商联、各人民团体和各界人士，向人民解放军、武警驻通部队官兵、公安干警和部、省驻通单位，向所有关心和支持南通发展的海内外朋友，致以崇高的敬意和衷心的感谢！

　　同时，我们也清醒看到，全市经济社会发展中还存在不少矛盾和问题，经济发展面临的深层次、结构性矛盾依然存在，自主创新能力和产业竞争力还不强，服务业占比还较低，扩量与转型的任务仍然艰巨；要素配置偏紧对经济增长压力增大，资源瓶颈制约更加突出，部分中小企业生产经营困难，财政减收增支因素增多；城市功能还需加快提升，出行难、停车难等问题仍然存在；社会建设体系还不够完善，城乡居民增收的增长点还不多，部分群众生活仍较困难，保障和改善民生的任务仍然繁重，维护社会稳定仍有很多工作需要深入做好。同时，政府自身建设中还存在一些薄弱环节，有的部门服务效能还不够高，工作中推诿扯皮等现象还时有发生，少数政府工作人员甚至以权谋私。对这些问题，我们将认真对待，切实解决。

　　二、2012年目标任务

　　2012年是党的十八大召开之年。新的一年，既面临难得机遇，两大国家战略向纵深推进，省第十二次党代会明确提出“支持南通建设长三角北翼经济中心”，政策导向效应进一步释放，进入桥港新时代的南通比较优势更加凸显；也面临更加严峻复杂的国内外环境，短期因素与长期因素、经济因素与非经济因素交织，组织、推进工作的难度更大。我们必须坚定必胜信心，以强烈的责任意识抢抓一切发展机遇；我们必须保持清醒头脑，以强烈的忧患意识推动各项工作再上新台阶，努力开创科学发展新局面。

　　今年政府工作的总体要求是：以邓小平理论和“三个代表”重要思想为指导，深入贯彻落实科学发展观，牢牢把握主题主线，全面落实中央和省经济工作会议以及省市党代会、市委十一届二次全会精神，按照“把握总基调、强化主抓手、突出着力点、营造好环境”的要求，围绕率先基本实现现代化和建设“一中心三城市”的奋斗目标，抓住机遇，奋发有为，坚定不移稳增长扩规模，更大力度抓创新调结构，更加注重抓开放促转型，更高层次重统筹强协调，全力以赴惠民生增和谐，落实“八项工程”、实现“八个领先”，以优异成绩迎接党的十八大胜利召开。

　　今年全市经济社会发展主要预期目标是：地区生产总值增长11%左右；地方一般预算收入增长15%以上；固定资产投资增长18%，社会消费品零售总额增长17%，外贸进出口总额增长10%；城镇居民人均可支配收入、农村居民人均纯收入均增长13%以上；居民消费价格指数涨幅低于省控目标；城镇登记失业率控制在4%以内；全社会研发投入占地区生产总值比重突破2.2%；万元地区生产总值能耗降幅和主要污染物减排完成省下达任务。

　　实现上述目标，我们将坚持“稳中求进、好中求快、又好又快”，着眼于“稳”，立足于“进”，致力于“抢”，突出于“快”，体现于“好”，着力做好以下十个方面工作：

　　（一）千方百计稳增长，推动经济综合实力再上新台阶。全面落实稳增长扩规模的各项措施，加强形势研判，注重政策创新，全力保持经济平稳较快发展。

　　保障要素、优化服务稳增长。强化信贷激励引导，确保全年贷款增量高于上年，增幅高于全省平均水平，促进存贷比明显提高；鼓励金融机构创新金融产品，扩大中小企业集合债券、短期融资券、中期票据、集合票据等发行规模；支持金融机构扩大表外业务规模，引导企业使用多种融资工具，新增上市企业3家以上；做大非营利性担保机构，健全担保体系，加大对中小企业贷款政策性担保扶持力度。创新思路，多措并举，帮助企业化解用地、用工、用电等要素制约。为实体经济发展创造良好环境，完善支持重点行业、中小微企业发展的政策措施，加强中小企业服务体系建设，发挥行业协会商会作用，提供高质量、高效率服务。

　　加大投入、做强企业稳增长。坚持以扩大有效投入保增长、促转型，重点抓好总投资4500亿元的150个市级重大项目建设。突出加大生产性投资，力争新投产总投资超5亿元产业项目超过40个，培育更多新的经济增长点。加快推进7个总投资超50亿元、超百亿元项目建设，开工建设5个总投资超50亿元项目，争取形成更多实物工作量。做好6个百亿元级项目前期工作，争取尽快开工建设。实施“规模培育千企升级工程”，新增一批大企业（集团）和“小巨人”企业。

　　稳定外贸、扩大消费稳增长。落实和完善出口退税、信保信贷等外贸扶持政策，推动企业保订单、多出口；安排专项资金，加快国家级船舶出口基地建设，积极申报国家级家纺外贸转型示范基地，扶持船舶、光伏等重点行业和企业巩固传统市场，开拓新兴市场；增强口岸功能，采取更多贸易便利化措施，扩大自营出口规模；提升外贸品牌化、特色化水平，提高自主知识产权和自主品牌出口产品比重，加快培育一批出口超亿美元企业、超10亿美元特色产业板块。着力扩大消费需求，积极培育旅游休闲、文娱健身、家政服务等新的消费热点，稳定房地产等带动力强的消费，扩大农村消费规模，提高消费对经济增长贡献率。

　　（二）奋力主攻重点，推动江海联动开发新突破。围绕“实现沿海开发全省领先”，深化功能分区规划，用足用好用活沿海开发、江海联动优势。

　　在港口功能和通道建设上实现突破。实施总投资超70亿元的10个重大港口项目，全市港口货物吞吐量力争超过1.9亿吨。推进洋口港区15万吨级进港航道试挖工程、吕四港区10万吨级挖入式港池、通州湾起步工程等项目，深化沿海港区30万吨级航道等前期研究工作。抢抓长江-12.5米深水航道上延机遇，加快沿江岸线整合和港口功能调整。推进现代综合交通网络建设。加快实施临海高等级公路、通洋高速、宁启铁路复线电气化改造、连申线航道整治等项目，争取沪通铁路、宁启铁路二期早日开工。开通运营海洋铁路，力争南通汽车客运枢纽东站主体工程完工。做好崇海大桥、海启高速、通苏嘉城际铁路、城市轨道交通等前期工作。加强沿海地区电力基础设施建设。

　　在园区经济和载体建设上实现突破。着力推进接轨上海、跨江合作、产城融合，大力发展园区经济，以一流的载体集聚一流的要素。强化省级以上开发区功能开发和特色园区培育，提高产业集聚度和综合承载力。争取1-2家省级开发区升格为国家级，新设1-2家省级开发区。推动苏通科技产业园、锡通科技产业园等跨江合作园区高端化、集聚化发展，引进一批标志性、品牌性项目。加快完善滨海园区配套功能，下决心发展重装备制造业。

　　在滩涂开发和体制创新上实现突破。加快推进4个区域滩涂围垦重点项目，确保完成7万亩。强化“沿海开发一盘棋”意识，完善市级统筹、市县联动、共建共享新机制。创新沿海开发融资模式，完善政策体系，促进各类要素向沿海前沿区域集聚。

　　（三）大力提升产业层次，增创转型升级新优势。深入落实转型升级工程，加快构建具有南通特色的现代产业体系。

　　突出高端化，提升发展主导产业。推动主导产业向产业链、价值链高端攀升，确保规模以上工业产值突破1万亿元。推动信息化与工业化深度融合，培育一批“两化融合”示范企业。加快提升船舶产业核心部件配套能力。提高电子信息产业发展水平，重点发展软件、光通讯、集成电路、新型电子元器件等产业。拉长纺织服装产业链条，加强研发基地建设，增强与文化创意、时尚休闲等产业融合发展能力，提高产业附加值和竞争力。

　　突出基地化，着力发展新兴产业。以培育型、成长型新兴产业特色基地为抓手，深入实施新兴产业发展“310”工程，打造10条特色产业链，培育新材料、生物医药、光电产业园等一批特色产业园区，确保新兴产业产值达到2350亿元，新增市级以上特色产业基地10家。提高海洋工程总装集成能力；拉长延伸新能源产业链，突破风电整机制造等关键环节；大力发展化工新材料、纺织新材料、合金新材料等产业；聚焦政策支持龙头企业、重点项目，新增营业收入超亿元新兴产业企业20家。

　　突出集聚化，加速发展现代服务业。实施现代服务业提速发展三年行动计划，重点发展“十大产业”，加快建设41个市级以上现代服务业集聚区，确保服务业增加值占地区生产总值比重突破40%。大力发展现代物流业，重点抓好港口综合型、货运枢纽型、市场配套型物流园建设，力争引进1-2家国际国内品牌物流公司和商品交易经纪公司，加快建设长三角北翼现代物流中心。加速做大旅游休闲业，推进“五山”地区整体开发和濠河景区水平提升，争取实现国家5A级景区和旅游度假区零的突破，加快建设江海旅游门户城市。支持发展金融业，力争增加值突破200亿元。提升发展商贸流通业，优化商贸布局，丰富商贸业态。推进制造业主辅分离，新增分离企业100家。加大服务业项目推进力度，力争服务业投资突破1000亿元。

　　（四）突出创新驱动，构筑人才新高地。落实科技创新工程，以创新驱动转型升级、人才引领产业发展，增强区域创新能力。

　　推动创新体系再优化。推进国家创新型试点城市建设，构建具有南通特色的技术创新体系。突出应用研发，完善鼓励技术创新和成果转化的政策体系、激励机制，全社会研发投入突破100亿元。突出企业主体，大力培育产业链骨干企业技术中心、产业技术创新战略联盟、技术标准联盟，新增省级以上企业技术中心6家；大力培育高新技术企业，高新技术产业产值占规模以上工业产值比重达到38%。突出开放创新，与国内外知名大学建立多方位、系统性的紧密合作关系，实施一批产学研合作项目。突出科技与金融融合，健全科技金融服务体系，大力发展创业投资、风险投资、私募股权投资等机构。

　　推动创新人才再扩容。把人才优势作为科学发展的第一优势，以更大力度实施“江海英才”计划，全面启动“312”产业人才发展行动计划，力争引进国家“千人计划”人才5名，省“双创计划”人才（团队）30名。花更大代价引进领军人才和创新团队，地方一般预算收入的3%用于科技人才发展。强化与高等院校、科研院所合作，瞄准重点地区，帮助企业引进人才，新增一批省级院士工作站，引进柔性人才1000名。建设更富竞争力的人才特区，完善人才工作体制机制和政策服务体系，为高层次人才提供一流的宜居创业环境。

　　推动创新平台再提升。以省级以上开发区为重点，全面推进“三创”载体建设，争取南通高新区升格为国家级、省级海安高新区获批。高标准建设南通科技园、国际软件园等创新园区，引进品牌企业，形成集聚带动效应。健全各类科技公共服务平台。

　　（五）坚定不移推进改革开放，增强科学发展新活力。坚持以改革破难题、促发展，以开放促改革、促创新，敢于先行先试，创新体制机制，让开放城市更具开放优势。

　　不断深化重点领域和关键环节改革。围绕加快转型升级，落实国家推进财税、金融、价格等改革的系列政策。围绕加强社会建设，抓好公立医院改革试点，深化文化事业单位改革。围绕城乡一体化发展，推进经济发达镇扩权强镇试点工作。围绕提升资源配置水平，深化国有资产管理体制改革，整合优化国有资产经营平台，形成参与城市建设、产业转型、企业重组合力；大力推动资源资产化、资产资本化、资本证券化，增强资本运作能力。

　　大力提升开放型经济国际化水平。提高利用外资水平。突出基地化、专业化招商，大力招引旗舰型、龙头型项目，力争引进超亿美元项目20个；大力提升新兴产业、现代服务业利用外资比重；更加注重引资金与引技术、引智力、引业态结合，大力招引各类研发中心、采购中心等地区性总部。更大力度发展服务外包产业，建设一批服务外包基地，做强船舶、家纺设计等产业，突破电子商务、云端服务等领域。提高外经合作水平。引导优势行业和重点企业到境外开展对外直接投资、国际工程总承包，并购研发机构、营销网络和知名品牌。提高口岸开放水平。编制全市海关特殊监管区布局规划，努力在综合保税区、保税港区等方面求突破。加强外事、侨务、港澳事务、对台事务等工作。

　　加快推动民营经济转型升级。毫不动摇大力发展民营经济，出台加快民营经济转型升级意见，以实施“三名”工程、“六大计划”为抓手，突出推动创业，进一步放开民资准入领域，降低准入门槛，掀起新一轮全民创业热潮；突出培大扶强，壮大一批百亿元级产业板块，新增一批营业收入超50亿元民营企业，继续建设全国一流建筑强市，建筑业施工总产值超过3500亿元；突出品牌培育，争创省级以上名牌产品75个，驰名、著名商标25件，创建全国知名品牌示范区2个。

　　（六）统筹城乡发展，实现农村面貌新改观。坚持“三化”互动并进，突出“民富村美风气好”新农村建设，加快城乡一体化发展。

　　落实农业现代化工程。毫不放松做好“三农”工作，积极构建现代农业体系。扩大现代高效农业规模。调优农业结构和布局，大力发展高效设施农业、外向农业、品牌农业和都市休闲农业，新增高效农（渔）业面积32万亩，其中，设施农（渔）业10万亩；建成市级以上现代农（渔）业园区17个，新增农产品加工集中区4个，抓好江海台湾农民创业园建设；加强农业标准化生产和农产品质量安全工作。提高农业市场化、科技化、组织化程度。加强农业社会化服务和科技推广体系建设，提高农业市场竞争力和科技贡献率。以龙头企业、农民专业合作组织为重点，加快培育现代农业生产经营主体，新增市级以上农（渔）业产业化龙头企业25家，农村三大合作组织农户入社率超过60%。改善农村发展环境。加大“四位一体”环境长效管理力度；探索具有南通特色的村庄环境整治路子，确保66%的行政村通过省级验收。

　　加强水利和农田基础设施建设。组织编制并实施全市水利现代化建设规划，完成投入25亿元。加快推进一批中小河流治理和重点水利工程，提高防洪除涝标准，增强防灾减灾能力；大力实施小型农田水利重点县（市）、大中型灌区等建设，加大中低产田改造力度，新建高标准农田20万亩，提高农业综合生产能力。

　　加大城乡一体化发展推进力度。推动城乡规划、产业布局、基础设施、公共服务、就业保障、社会管理等一体化发展。强化规划引导作用。加快推进县（市）城、乡镇总体规划修编，调整优化村庄布局规划，完善城乡空间布局。健全政策体系和制度框架。有序推进农村土地、户籍等制度改革，稳妥推进“万顷良田试点工程”和农民集中居住区建设，促进工业向园区集中、人口向城镇集中、居住向社区集中、土地向适度规模经营集中。创新农业农村投融资政策，鼓励金融机构向县（市）、乡（镇）延伸服务，吸引“三资”加快投入农业现代化建设。加快公共服务向农村延伸。抓好区域供水和农村饮水安全工程，全市城乡居民普遍饮上优质长江水；推进城乡客运一体化工程，村级通公交率达到75%；推进农村社区服务标准化建设，完善村级服务中心功能。

　　提高新型城镇化水平。优化城镇规划布局，统筹推进中心城市与县（市）城、重点中心镇建设，全市城市化率达到58.5%。支持各县（市）城按照建成区面积达到100平方公里的目标，加快向中等城市发展，增强城市功能。推动一批重点镇按照建成区面积达到30平方公里的目标，加快向小城市发展，促进产业和人口、资源等要素迅速集聚。按照“港城一体、区镇合一”的思路，强化吕四、长沙、三余等8个沿海前沿镇建设，打造特色鲜明的现代化滨海城镇带。

　　（七）增强集聚辐射功能，展现中心城市新魅力。加快中心城市建设，大力实施“繁荣、提升、整合、扩容、解难、服务”六项措施，提高中心城市首位度，增强市民荣誉感和归属感。

　　优化城市空间形态。以规划引领空间布局。深化“1+5+8”中心城市空间布局规划，细化新城区、观音山新城、北翼新城等区域扩容规划，推动带状城市向组团城市发展，加快形成一核三片区的“T型城市组团”。以规划引领功能分区。对重点区域进行整合，系统规划中心城市商务、商业、文化、金融、教育、卫生等布局，促进各类功能相对集聚。以规划引领城市风貌。强化城市特色空间引导，优化建筑、水系、绿化等景观设计，彰显时尚秀美、精致协调的“江风海韵北上海、中国近代第一城”魅力。

　　强化城市交通体系建设。以规划引领交通架构，深化城市高速路网、铁路网，市区快速路网、轨道交通网等规划和老城区交通改善及重要交通节点改善方案研究，综合考虑各种交通体系的衔接配套及交通枢纽建设，引导城市空间结构调整。深入推进市区“102030”交通畅通工程，实施江海大道东延、西延和东方大道快速化改造等8个快速路项目。推进工农路北延、通富路北延等49项骨干道路工程以及36项支路和重要节点工程，密切城市组团间交通联接。着力缓解老城区出行难、停车难，疏通路网，改善节点，多级分流，提高组织化水平。大力实施公交优先战略，新增运营线路12条；市区继续推进一批公共停车设施建设；加强出租车营运管理，提高行业服务水平。

　　加大重点区域建设改造力度。着眼“提高品质、凸显内涵、打造亮点”，推进总投资203亿元的146个重点城建项目，推动重点区域由形态开发向功能开发转变。加快以新城区为重点的城市核心区建设。促进新城区繁荣，积极发展以金融、商务、科技等为主体的城市经济、服务经济，加快金融街区建设和社会服务设施配套，促进各类要素加速向新城区集聚；抓好观音山新城起步区建设，全面规划商业服务、文化体育、教育卫生等功能布局。优化市区滨江、通吕运河、海港引河等区域景观。大力推动老城区改造提升，结合历史文化街区保护改造，布局一批高端文化、休闲、商务等业态。强势推进三大片区建设。以开发区能达商务区、苏通科技产业园为重点，推进一批总部楼宇、城市综合体、特色商业街区等建设，实现多种功能融合发展，加快建设富有区域特色的现代化南翼新城。推动港闸区火车站站前区、站北区和上海市北高新（南通）科技城三大板块建设，加快建设北翼新城。推动通州区加快融入主城区，深化研究金沙城区、先锋地区、滨海新区等功能板块的发展方向与规划，加快建设东部新城。

　　提升城市科学化管理和为民服务水平。开展新一轮市容环境综合整治。以“精致、细腻、整洁、有序”为目标，破解城市管理难题，打好户外广告整治等“五大攻坚战役”，实施建筑物出新改造等“五大提档工程”，让城市变得更靓丽。强化城市网格化改造和管理。网格式、街坊化推进30个城中村改造和27个老小区整治。加强小区物业管理。发挥数字化城管平台作用，实现网格化、精细化、智能化管理。健全“二级政府、三级管理、四级网络”长效管理体制，构建“大城管”工作格局。完善城市公共服务设施。规划布局一批小游园，为市民创造更多绿色休憩空间；加强社区文化、体育等公共设施建设；实施崇海水厂等23个公用事业项目，优化城市水电气服务体系，完成市区天然气置换。

　　（八）加快建设文化强市，促进城市软实力新提升。落实文化建设工程，以开放的姿态推动文化大发展大繁荣，加快建设江海特色文化强市。

　　充分发挥先进文化引领作用。建设社会主义核心价值体系，弘扬“三创三先”江苏精神和“包容会通、敢为人先”南通精神，营造昂扬向上的社会氛围；鼓励见义勇为，弘扬凡人善举，光大精神文明“南通现象”；突出以诚信为龙头的思想道德建设，在全社会倡导公正、责任、诚信的价值取向。健全文明城市创建长效机制，推动更高水平的文明城市建设。

　　增强公共文化服务能力。构建覆盖全市、惠及全民的公共文化服务体系，为群众提供更多公共文化产品。大力推进文化惠民工程，加快市图书馆新馆、报业新闻传媒中心等一批文化设施建设，推动各县（市）区公共图书馆、文化馆建设和达标改造，实施乡镇（街道）综合文化站、村文化室达标工程和“农家书屋”提优工程、乡镇广播电视服务站标准化工程，完成行政村体育健身设施提档升级。大力推动文化产品生产，传承“中国近代第一城”优秀文化，努力推出一批群众喜爱、体现时代风采、展示地域文化特色的精品力作。大力加强历史文化保护，进一步放大环濠河博物馆群文化资源优势，做好创建全国公共文化服务示范项目实施工作，推进一批历史文化保护工程，积极传承优秀民俗艺术、非物质文化遗产，彰显国家历史文化名城新风采。

　　着力提高文化产业竞争力。着力构建现代文化产业体系，加快把文化产业培育成支柱产业。以实施重点项目引领规模化发展，突出创意设计、新兴媒体、会展文博、印刷包装等发展潜力大的领域，推进总投资500亿元的150个文化项目建设。以壮大地方传统、优势产业推动特色化发展，做强广播影视、文艺演出等传统优势产业，加快培育工艺美术、民间工艺等特色产业。以搭建载体促进集约化发展，着力整合资源、强化创新，加快建设一批富有竞争力的文化产业集聚区。

　　（九）强化社会建设和管理，形成民生和谐新局面。落实民生幸福和社会管理创新工程，坚持以人为本，突出以改善民生为重点构建社会建设“十大体系”，完善社会管理体系，更好服务百姓。加大民生投入，确保一般预算支出用于民生比重高于上年。

　　深入实施居民收入倍增计划。落实“就业、创业、产业、物业”等富民举措，努力实现居民收入增幅全省领先。扩大就业增加工资性收入。增强乡镇（街道）和村（社区）公共就业服务能力，突出抓好高校毕业生、城镇就业困难人员、农村富余劳动力等重点群体就业工作。继续提高最低工资标准，推进工资集体协商，确保企业职工工资增长基准线与经济增长同步提高。实施事业单位绩效工资。促进创业增加经营性收入。把以创业带动就业作为促进增收的主攻方向，大力实施“万人创业计划”，努力营造鼓励脚踏实地、勤劳创业、实业致富的社会氛围。鼓励投资增加资产性收入。推动多种形式的股份合作，支持投资服务机构和理财机构发展，鼓励居民依法开展投资经营活动。强化扶持增加转移性收入。继续对生活困难群众实施价格上涨动态补贴，做好扶贫和老区开发工作。坚持以强村带动富民，落实扶持村级集体经济发展各项政策，促进行政村集体收入较快增长。切实搞好物价调控监管，加强“菜篮子”重点工程和蔬菜保供基地建设，着力保持物价总水平基本稳定。

　　健全基本公共服务体系。围绕“学有优教”，加快实施学前教育普及提升工程，促进义务教育优质均衡发展，推动普通高中优质特色发展，创新发展职业教育，提升高等教育内涵建设水平，加强社会教育、特殊教育；强化校舍、校车安全工作；实施通大附中、市二中新校区等重点项目。围绕“应保尽保”，巩固新农保全覆盖成果，实现城乡居民社会养老保险全覆盖，全面实施城镇职工基本医疗保险和生育保险市级统筹；市区符合条件的被征地农民全部纳入城镇企业职工基本养老保险。进一步提高城乡低保等保障水平，动员各方面力量参与社会救助。围绕“病有良医”，提高新农合筹资标准，新农合县乡两级政策范围内住院补偿比例超过70%；开展基层医疗卫生服务机构标准化创建，完善农村三级医疗卫生服务体系，巩固和规范基本药物制度，强化公共卫生均等化服务，让群众看病更方便、负担能减轻。优化计划生育服务。围绕“住有宜居”，系统推进拆迁安置房、保障性（限价）商品房、公共租赁房、廉租房和经济适用房建设。围绕“老有颐养”，适应人口老龄化趋势，逐步完善社会养老体系，健全市县镇村四级养老服务设施，乡镇（街道）居家养老服务中心建成率超过50%。做好关心下一代、法律援助、红十字、慈善等工作。

　　全面提升社会管理科学化水平。巩固社会管理创新领先成果，争创全国社会管理创新示范城市。完善社会管理制度。健全群众利益维护、社会稳定风险评估、社会矛盾纠纷大调解等制度。深化公共安全服务、社会诚信、实有人口服务管理等体系建设。加强和改进新形势下信访工作。加强社会管理平台建设。构筑新型社会管理服务平台，启动人口基础数据库建设，推动管理主体社会化、标准精细化、流程法治化、手段信息化。强化基层基础建设。推行村居（社区）“一委一居一站一办”管理服务模式，推进村级“四有一责”建设，力争65%的城市社区和55%的农村社区达到省级和谐社区建设标准。发展壮大专业社工队伍，大力培育社团组织。支持人民团体发挥密切联系群众的桥梁作用，加快工青妇活动中心等项目建设。深化长安南通创建。健全现代治安防控体系，严密防范、依法打击各种违法犯罪活动。完善公共突发事件应急管理机制。加强网络信息管理。严格落实安全生产责任制，强化食品药品安全监管。全面实施“六五”普法规划。加强国防动员和后备力量建设，做好民族、宗教、双拥等工作。

　　（十）深化生态文明创建，培育绿色发展新特色。落实生态文明建设工程，打造一流生态环境，力争建成江苏江北首个国家生态市和国家环保模范城市群。积极发展绿色低碳经济。构建绿色产业链和资源循环利用链，在化工、印染等重点行业实施清洁生产和循环经济项目各100个。推进工业园区生态化，建设一批绿色园区。着力发展绿色建筑。加快低碳技术推广应用。坚定不移推进节能减排。从严把好项目能评和环评审查关，大力淘汰落后产能。推进城镇生活污水处理厂和配套管网建设，实施污水处理厂提标改造和三大电厂脱硝工程。落实耕地保护、节约用地制度和水资源管理制度。切实加强生态环境建设。推进新一轮“绿色南通”建设，实施一批园林景观和道路绿化工程，市区新增绿地200公顷以上；加大植树造林力度，全市新增造林面积16.8万亩。深入实施蓝天、清水工程，加大秸秆禁烧和综合利用力度，做好PM2.5监测工作，空气质量优良天数力争比上年增加；做好应急水源地建设、饮用水源地保护和引江调水工作，推进城乡河道整治，努力实现“水通、水动、水清、水安、水灵、水美”。加强重点领域环境治理，集中整治损害群众利益的环境违法行为。

　　三、加强政府自身建设

　　率先基本实现现代化对政府自身建设提出新的更高要求。我们将牢固树立“作风就是生产力、效能就是竞争力”和“政务就是服务、公务员就是服务员”的理念，问政于民、问需于民、问计于民，加快转变政府职能，努力建设人民满意的服务型政府。一是坚持执政为民，进一步增强政务服务竞争力。着力构建具有南通独特竞争力的服务品牌，树立“效能南通”形象，把执政为民的要求落到实处。以争创全省最佳办事环境为抓手，巩固行政审批“三集中、三到位”成果，把政务中心建成“政府服务群众的第一窗口”，使南通成为行政效率的高地、行政成本的洼地。充分发挥“12345”政府服务热线等“五位一体”民生诉求表达服务机制作用，加快形成从村（社区）到市的四级便民服务网络。集中力量为民兴办10件42项实事。二是坚持勤政高效，进一步提升行政执行力。以省、市党代会目标任务落实的成效检验执行力，目标化、指标化、标识化、项目化落实各项任务，确保实现全年预期目标。以良好的状态提升执行力，解放思想，敢于担当，进一步形成想发展、敢发展、会发展的生动局面。以完善的机制保证执行力，强化重大决策、重点工作、重要项目的推进、协调和跟踪督查，确保政令畅通。以高效快干的作风体现执行力，切实做到政策落实快、项目推进快、矛盾处理快。三是坚持依法行政，进一步提升政府公信力。完善重大事项听证和决策跟踪反馈、评估制度，提高决策的科学化、民主化、法治化水平。加大行政权力公开透明运行、行政审批公开、政府信息公开力度，让行政权力始终在阳光下运行。探索建立“首查教育、二查整改、整改到位不处罚”制度，大力推进联合执法、便民执法和行政执法情况通报制度。自觉接受人大及其常委会的法律监督、工作监督和政协的民主监督，高度重视办好人大代表议案、政协建议案和人大代表建议、政协提案。认真听取各民主党派、工商联、无党派人士和各人民团体的意见，主动接受社会各方面的监督。四是坚持清正廉洁，进一步增强政府系统凝聚力。推进政府系统惩防体系建设，健全重点领域、重点部门、重点资金使用和重点项目审计制度，严格执行廉洁从政各项规定，有效预防、坚决惩治腐败。巩固和深化专项整治成果，继续加强公务接待、公务用车、公费出国出境等管理，推进政府系统厉行节约。加大效能监察、绩效考评和行政问责力度，切实防止行政不作为、慢作为、乱作为。强化政府系统干部的教育管理和作风建设，求真务实，深入基层，加强调研，倾听民意，让“科学发展向上攀登、联系群众向下扎根”成为政府系统党员干部的不懈追求，努力建设一支“讲党性、有激情、能干事”的公务员队伍。

　　各位代表，南通正处于创新驱动、转型发展的重要关头，我们深感责任重大、使命光荣。让我们紧密团结在以胡锦涛同志为总书记的党中央周围，在省委、省政府和市委的坚强领导下，同心同德、开拓创新，抢抓机遇、埋头苦干，为南通落实“八项工程”，实现“八个领先”，在江苏江北率先基本实现现代化而努力奋斗，以优异成绩迎接党的十八大胜利召开！
